# Methicillin resistant *Staphylococcus aureus* causing osteomyelitis in a tertiary hospital, Mwanza, Tanzania

**DOI:** 10.1186/s13018-020-01618-5

**Published:** 2020-03-05

**Authors:** Vitus Silago, Martha F. Mushi, Boniface A. Remi, Alute Mwayi, Stephen Swetala, Conjester I. Mtemisika, Stephen E. Mshana

**Affiliations:** 1 Microbiology and Immunology department, Weill Bugando School of Medicine, Catholic University of Health and Allied Sciences, P. O. Box 1464, Mwanza, Tanzania; 2 Department of Surgery, Bugando Medical Centre, P. O. Box 1370, Mwanza, Tanzania; 3 Central Pathology Laboratory, Bugando Medical Centre, P. O. Box 1370, Mwanza, Tanzania

**Keywords:** Staphylococcal osteomyelitis, Methicillin resistant *S. aureus*, Bugando Medical Centre, Mwanza, Tanzania

## Abstract

**Background:**

Culture results of fluid/pus from sinuses or open wound are not reliable in establishing the causative agent of osteomyelitis due to the high chances of contamination of superficial contaminants. Bone fragments obtained during surgery have been recommended as ideal sample to establish pathogens causing osteomyelitis. This study investigated pathogens causing osteomyelitis among patients undergoing orthopedic surgical treatment at Bugando Medical Centre.

**Methods:**

A cross-sectional hospital-based study was conducted from December 2017 to July 2018 among 74 patients with osteomyelitis who underwent surgical treatments at Bugando Medical Centre, Mwanza, Tanzania. Bone fragments were collected using sterile 10 ml of in-house prepared brain heart infusion broth (Oxoid, UK) during surgery. Specimens were processed according to standard operating procedures within an hour of collection. Data were analyzed using STATA 13.0.

**Results:**

The median age of study participants was 12 with inter quartile range of 8–20 years. The majority 45 (60.8%) of participants were male. All 74 non-repetitive bone fragment specimens had positive culture, of which 17 had dual growth of bacteria resulting to 91 bacterial isolates. Out of 91 isolates, 63 (85.1%) were *Staphylococcus aureus* (*S. aureus*) of which 18 (28.6%) were confirmed to be methicillin resistant *Staphylococcus aureus* strains. Fever was significantly associated with Staphylococcal osteomyelitis (100% vs. 79.6%, *p* = 0.029).

**Conclusion:**

About one third of cases of Staphylococcal osteomyelitis in the current study were caused by methicillin resistant *Staphylococcus aureus*. There is a need of tailoring antibiotic management of osteomyelitis based on culture and sensitivity results for the better treatment outcome of the patients.

## Introduction

Osteomyelitis is an inflammation of the bone that can be localized to only one part of the bone such as bone marrow, periosteum, or cortex [1]. In few occasions , osteomyelitis can disseminate to the surrounding tissues [1]. Osteomyelitis can be classified by the location within the bone, extent of dissemination and source of infection [1]. Bacteria are the commonest cause of osteomyelitis with *S. aureus* implicated in more than 75% of cases. *S. aureus* spreads to the bones through blood (hematogenous osteomyelitis) or directly as a result of open fractures [2, 3].

Staphylococcal osteomyelitis is a major global health concern because of the increasing antimicrobial resistance. Treatment of osteomyelitis is always complex because it requires a well-coordinated team of radiologist, orthopedic surgeon, and infectious diseases specialist [4]. Culture of fluid/pus from sinuses or discharging ulcers is not reliable, thus leading to the dependence of imaging and high suspicion of a surgeon in managing osteomyelitic patients. To determine the causative pathogen implicating in osteomyelitis, a sample of choice is bone fragments because culture of fluid/pus from sinuses or open is always associated with superficial bacteria contaminants [4, 5]. This study investigated the pathogens causing osteomyelitis among patients undergoing surgical treatment at Bugando Medical Centre, Mwanza-Tanzania for the purpose of generating data that can be used to devise antibiotic treatment guidelines.

## Methodology

### Study design, participants, duration, setting and specimen collection

It was a cross sectional hospital based study, conducted from December 2017 to July 2018 among 74 patients with osteomyelitis admitted for surgery at Bugando Medical Centre (BMC), Mwanza, Tanzania. BMC is a tertiary referral hospital with 1000 bed capacity, serving seven regions namely: Mwanza, Musoma, Simiyu, Shinyanga, Kagera, Kigoma, and Geita. The study included all patients with clinical diagnosis of osteomyelitis planned for orthopedic surgery. As part of routine clinical care at enrollment, body temperature was measured using a digital thermometer MDD 93/42/EEC, “0197” (Holding Corp. GmbH Hamburg) and all patients with body temperature above 37.5^°^ C were termed as febrile [6]. During surgical procedures, bone fragments were collected and placed into sterile universal bottle containing 10 ml of in-house brain heart infusion (BHI) broth (Oxoid, UK) [7] to increase the yield of pathogenic bacteria. Specimens were sent to Microbiology laboratory of the Catholic University of Health and Allied Sciences within 1 h of collection.

### Laboratory procedures

In the laboratory, universal bottles with samples were gently shaken 10 times, and then incubated at 37^°^ C for 6 h [7]. After 6 h of incubation, specimens were mixed gently and a loop full (10 μl) was inoculated on sheep blood agar and MacConkey Agar followed by aerobic incubation at 37^°^ C for 24 h. Conventional biochemical identification tests: Gram stain, catalase and coagulase, DNase were used to identify Gram-positive bacteria while Triple sugar iron agar (TSI), Sulfur-Indole-Motility (SIM), Simmons citrate, urease, and oxidase were used to identify Gram-negative bacteria [8]. Antibiotics susceptibility testing was performed using Kirby-Bauer disc diffusion technique. Tetracycline 30 μg, gentamicin 10 μg, ciprofloxacin 5 μg, clindamycin 2 μg, and vancomycin 30 μg were used for Gram-positive bacteria while ampicillin 10 μg, trimethoprim-sulfamethoxazole 1.25/23.75 μg, tetracycline 30 μg, gentamicin 10 μg, ciprofloxacin 5 μg, amoxycillin-clavulanic acid 20/10 μg, ceftriaxone 30 μg, ceftazidime 30 μg, piperacillin-tazobactam 100/10 μg, and meropenem 10 μg were used for Gram-negative bacteria [9]. Cefoxitin 30-μg discs were used for detection of MRSA as per Clinical Laboratory Standard Institute (CLSI) recommendations [10].

Briefly, pure fresh colonies were suspended in sterile 0.85% normal saline to make a suspension equivalent to 0.5McFarland. Using sterile cotton swab, suspension was inoculated on the entire surface of Muller Hinton agar (MHA) plate; thereafter, antibiotic discs were seeded within 15 min of inoculation. MHA plates were incubated aerobically at 37^°^ C for 18–24 h. Clinical and Laboratory Standards Institute (CLSI-2016) was used to interpret zones of inhibitions [10].

### Quality control

*Staphylococcus aureus* ATCC 25923 and *Escherichia coli* ATCC 25922 were used as control strains.

### Ethical considerations

Protocol of this study was ethically approved by the joint CUHAS/BMC research ethics and review committee and given certificate number 470/2017. All participants signed informed consent and those aged below 18 years their parents/guardians’ consented on their behalf. Laboratory results were timely reported to the attending physicians to guide rational antibiotics therapy.

## Results

### Socio-demographic and clinical characteristics of participants

A total of 74 osteomyelitic patients with median age of 12 and inter quartile range of 8–20 years were enrolled. Males made the majority 45 (60.8%) of the study participants. The majority 66 (89.2%) of participants had infections of the long bones. A total of 20 (27%) and 32 (43.2%) had fever and history of antibiotic use before the index admission, respectively. The median duration of antibiotic use was 2 weeks with inter quartile range of 2–4 weeks. The most commonly used antibiotic was ampicillin-cloxacillin which was reported in 65.6% (21/35) of patients (Table 1).

**Table 1:** Socio-demographic and clinical characteristics of study participants

Characteristics		Frequency (*N*)	Percentages (%)
Median age (IQR) in years		12	[8–20]
Sex	Males	45	60.8
	Females	29	39.2
Education level	None formal education	21	28.4
	Primary school	32	43.2
	Secondary school	15	40.3
	College and above	6	8.1
Referrals outside Mwanza	No	35	47.3
	Yes	39	52.7
Major complaint	Sepsis	32	43.2
	Trauma	42	56.8
Infected bone	Long bone	66	89.2
	Short bone	8	10.8
Infected site condition	Discharging	65	87.8
	Swelling	9	12.2
Fever during enrollment	Yes	20	27
	No	54	73
Pre-exposed to antibiotics	Yes	32	43.2
	No	42	56.8
Median duration (IQR) of drug use in weeks		2	[2–4]
Type of antibiotic exposed	Ampicillin-cloxacillin (Ampiclox)	21	65.6
	Ceftriaxone	5	15.6
	Ampiclox with ceftriaxone	4	12.5
	Herbal medicine	2	6.3

### Laboratory results

**Fig. 1:**
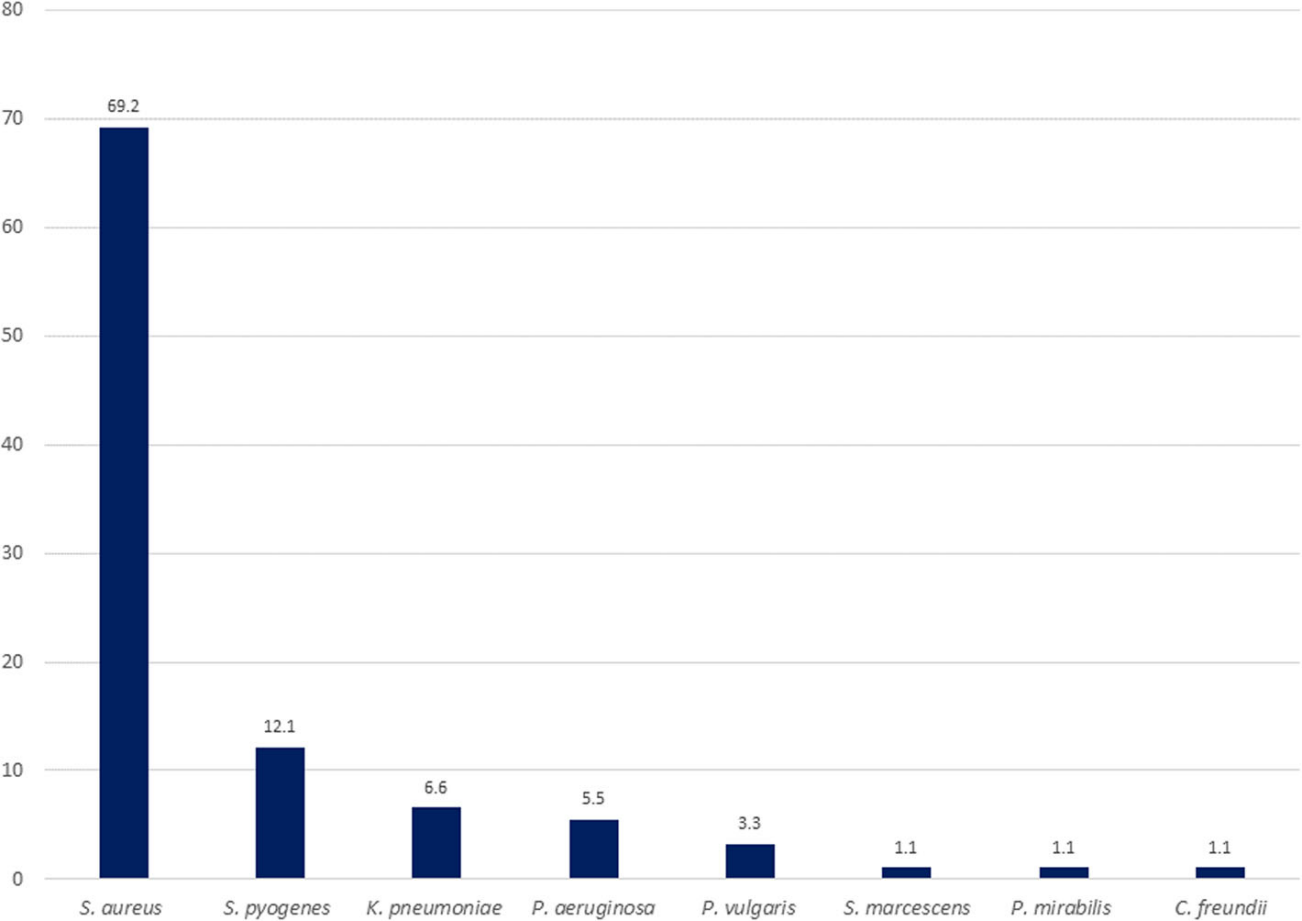
Bacteria isolated in 74 patients with osteomyelitis

All 74 non-repetitive samples yielded growth of pathogenic bacteria on culture. Seventeen samples had dual growth making a total of 91 isolates. The majority of isolates 74 (81.3%) were Gram-positive bacteria. Of 17 patients with dual bacterial growth, 11(64.7%) had *Streptococcus pyogenes* and *S. aureus*, 3(17.6%) had *Proteus vulgaris* and *Klebsiella pneumoniae*, and 3(17.6%) had *Pseudomonas aeruginosa* with either *Klebsiella pneumoniae, S. aureus*, or *Citrobacter freundii*. In general, the most frequently isolated bacteria was *S. aureus* 69.2% (63/91) followed by *Streptococcus pyogenes* 12.1% (11/91) and *Klebsiella pneumoniae* 6.6% (6/91) (Fig 1). Methicillin resistant *Staphylococcus aureus* (MRSA) strains were confirmed in 18 (28.6%) of 63 *Staphylococcus aureus* isolates (Table 2).

**Table 2:** Antibiotic susceptibility patterns of isolated bacteria

Antibiotic agents	Potency	Gram positive bacteria		Gram negative bacteria	
		Sensitive	Resistant	Sensitive	Resistant
Ampicillin	10 μg	NA	NA	0%	100%
Trimethoprim-sulphamethaxazole	1.25/23.75 μg	NT	NT	58.3%	41.7%
Tetracycline	30 μg	100%	0%	58.3%	41.7%
Gentamicin	10 μg	100%	0%	76.5%	23.5%
Ciprofloxacin	30 μg	100%	0%	100%	0%
Clindamycin	10 μg	98.6%	1.4%	NA	NA
Cefoxitin (*S. aureus* only)	30 μg	71.4%	28.6%	NA	NA
Vancomycin	30 μg	100%	0%	NA	NA
Ceftriaxone	30 μg	NA	NA	33.3%	66.7%
Ceftazidime	30 μg	NA	NA	35.3%	64.7%
Piperacillin-tazobactam	100/10 μg	NA	NA	64.7%	35.3%
Amoxicillin-clavulanic acid	20/10 μg	NA	NA	0%	100%
Meropenem	10 μg	NA	NA	100%	0%

*NT* not tested, *NA* not applicable

### Susceptibility patterns

Generally, Gram-positive bacteria were 100%, 100%, 98.7%, 100%, and 100% sensitive to gentamicin, ciprofloxacin, clindamycin, tetracycline, and vancomycin, respectively. Out of 63 *S. aureus*, 18 (28.6%) were MRSA with 10 of MRSA strains being isolated from patients with history of antibiotic use.

The proportion of resistance among Gram-negative bacteria was as follows: ampicillin (100%), amoxicillin-clavulanic acid (100%), ceftriaxone (66.7%), ceftazidime (64.7%), trimethoprim-sulfamethoxazole (41.7%), tetracycline (41.7%), piperacillin-tazobactam (35.5%), gentamicin (23.5%), ciprofloxacin (0%), and meropenem (0%) (Table 2).

### Factors associated with Staphylococcal osteomyelitis

By chi-square test, only having fever was statistically associated with Staphylococcal osteomyelitis (100% vs. 79.6%, *p* = 0.029) (Table 3). The sub-analysis by age (children vs. adults) found no significance difference terms of pathogens distributions and factors associated with Staphylococcus osteomyelitis (data not shown).

**Table 3:** Factors associated with Staphylococci osteomyelitis

Characteristics		Staphylococcal osteomyelitis		Chi^2^	*p* value
		Positive *n* (%)	Negative *n* (%)		
Age category	Children	44 (89.8)	5 (10.2)	0.963	0.124
	Adult	19 (76.0)	6 (24.0)		
Sex	Females	26 (89.7)	3 (10.3)	0.7699	0.380
	Males	37 (82.2)	8 (17.8)		
Referral outside Mwanza	No	30 (85.7)	5 (14.3)	0.0176	0.894
	Yes	33 (84.6)	6 (15.4)		
Education level of participant	Non-formal	19 (90.5)	2 (9.5)	2.5445	0.467
	Primary level	28 (87.5)	4(12.5)		
	Secondary level	12 (80.0)	3 (20.0)		
	College and above	4 (66.7)	2 (33.3)		
Complaint	Sepsis	28 (87.5)	4 (12.5)	0.2492	0.618
	Trauma	35 (83.3)	7 (16.7)		
Condition	Discharging	57 (87.7)	8 (12.3)	2.2615	0.097
	Swelling	6 (66.7)	3 (33.3)		
Fever during enrollment	Yes	20 (100.0)	0 (0.0)	4.7854	0.029
	No	43 (79.6)	11 (20.4)		

## Discussion

Dependence on imaging in the management of osteomyelitis in resource limited settings [11] is associated with poor prognosis of patients and morbidity such as amputation of infected bone. In the places where there is increased isolation of multidrug resistant bacteria, rational antibiotic therapy is mandatory for proper patients’ management [12]. However, in many resource limited settings, there is no routine culture for the diagnosis of osteomyelitis. Good prognosis of patients with osteomyelitis depends much on proper identification of etiological agent and timely treatmen t[13].

There is limited data regarding the pathogens causing osteomyelitis from developing countries. The majority of participants in this study were adolescent males, with infection of long bones as a result of trauma. The majority of these infections had purulent drainage from infection sites as previously reported elsewhere [14–16]. The escalating burden of long bones fracture in Tanzania among adolescent males is highly associated with the legalization of motorcycles as a means public transport [17, 18]. As previously reported [18–20], this study has confirmed that purulent discharge from the affected part of the bone is the commonest symptoms of osteomyelitis. The purulent discharge from the affected part of the bone increases the suspicious index of pyogenic infections and call for the need of microbiological investigations [21].

The 69.2% prevalence of the Staphylococcus osteomyelitis in the current study was certainly high. However, these results align with previous reports which reported *S. aureus* as a major pathogen causing osteomyelitis [22, 23]. It was observed that about one third of *S. aureus* isolates were MRSA which is almost double to what was observed 10 years ago in the same setting among *S. aureus* isolates from wounds [24, 25]. These findings cement the previous observations of increasing trend of MRSA in study settings made by Moremi et al*.* in 2016 [26].

Over the counter use of antibiotics and irrational empirical treatment of bacterial infections in the study settings [27] might result to the selection of resistant *Staphylococcus aureus* strains such as MRSA. This is further supported by the fact that in the current study the majority of patients with history of antibiotic use were using ampicillin-cloxacillin hence more likelihood of selecting MRSA strains.

The current study has observed that *S. aureus* isolates were highly susceptible to chloramphenicol, clindamycin, and vancomycin. This could be explained by the fact that these antimicrobial agents are not used in self-medication as penicillin, ampicillin, and amoxycillin. In addition, clindamycin and vancomycin are preserved as the second line treatment regimen [11]. The standard treatment guidelines in Tanzania recommend the use of ampicillin, tetracycline, and/or erythromycin in management of gram positive bacterial infection [11]. Based on these results, clindamycin should be considered as the first line empirical therapy while waiting for culture and sensitivity results, and when gram negative pathogens are suspected Piperacillin tazobactam can be added. These recommendations are supported by a previous systemic review [28].

Only fever was significantly associated with Staphylococcal osteomyelitis (*p* = 0.029). Fever is the commonest response of infection or inflammation whereby the interaction between exogenous pyogenic bacteria and the organum vasculosum of the lamina terminalis (OVLT) induces production of cytokines and increases synthesis of prostaglandin E2 (PGE2) resulting to body temperature rise [29].

## Limitation

Due to limited diagnostic facilities, the study did not investigate all range of pathogens that may cause osteomyelitis such fungi, *Mycobacteri*a spp., and anaerobic pathogens. In addition, data regarding involvement of the joint were not collected.

## Conclusion and recommendation

This study found high prevalence of Staphylococcal osteomyelitis among patients underwent surgical treatment at Bugando Medical Centre with a third of these patients infected with MRSA. Fever was statistically significant associated with positive Staphylococcal osteomyelitis. There is a need to tailor antibiotic management of osteomyelitis based on culture and sensitivity patterns for the better outcome of the patients.

## Data Availability

The data is available upon request and the request should be made to the Director of Research and Innovation, Catholic University of Health and Allied Sciences (vc@bugando.ac.tz).

## Abbreviations

BHI: Brain heart infusion
BMC: Bugando Medical Centre
CLSI: Clinical Laboratory Standard Institute
MHA: Muller Hinton agar
MRSA: Methicillin resistant *Staphylococcus aureus*
OVLT: Organum vasculosum of the lamina terminalis
SIM: Sulfur-Indole-Motility
TSI: Triple sugar iron agar
